# A Physics-Guided Dual-Sensor Framework for Bearing Fault Diagnosis in PMDC Motor Drives

**DOI:** 10.3390/s26041363

**Published:** 2026-02-20

**Authors:** Tae-Seong Sim, Nnamdi Chukwunweike Aronwora, Jang-Wook Hur

**Affiliations:** Department of Mechanical Engineering (Department of Aeronautics, Mechanical and Electronic Convergence Engineering), Kumoh National Institute of Technology, 61 Daehak-ro, Gumi-si 39177, Gyeonsangbuk-do, Republic of Korea; simteasog0417@kumoh.ac.kr (T.-S.S.); naronwora@kumoh.ac.kr (N.C.A.)

**Keywords:** bearing fault diagnosis, PMDC motor, envelope analysis, multi-sensor vibration signal, vibration transmission, physics guided features

## Abstract

Rolling-element bearing faults are a primary mechanical failure mode in rotating systems. In Permanent Magnetic DC (PMDC) motor applications operating under variable torque, vibration-based diagnosis is affected by load-dependent excitation and commutation-induced disturbances, which introduce amplitude bias and reduce the reliability of conventional statistical features. This study proposes Cross-Reference Energy Attention (CREA), a physics-guided dual-sensor feature framework for three-class bearing states in PMDC motor systems. CREA isolates fault-relevant content within a hardware-agnostic, empirically selected mid-frequency carrier band and incorporates a spatially separated reference sensor to evaluate transmission consistency. This design suppresses disturbances generated locally by the motor while retaining structurally transmitted bearing signatures. Experiments were conducted on a PMDC motor dynamometer with seeded bearing defects under controlled torque variation. GroupKFold cross-validation was implemented using the acquisition run as the grouping variable to prevent data leakage across runs. Under per-run normalization designed to eliminate amplitude memorization, conventional motor-side baseline features degraded to 0.495 ± 0.110 window-level accuracy, whereas the four-feature CREA representation maintained 0.999 ± 0.002. Systematic ablation and SHAP analysis demonstrate that carrier-band energy features provide the dominant discriminatory contribution, while cross-sensor interaction metrics supply complementary transmission validation consistent with the underlying mechanical model.

## 1. Introduction

The operational reliability of Permanent Magnet DC (PMDC) motor drives is critical in automated systems, including robotics, automotive subsystems, and industrial conveyors [1]. While these electromechanical systems are susceptible to various failure modes, rolling-element bearing degradation is the most prevalent source of unexpected mechanical failure [2,3]. Bearing faults in PMDC architectures must be detected early to maintain system availability and prevent secondary mechanical damage.

Vibration-based condition monitoring is the established method for bearing fault diagnosis [4]. Under relatively stable operating conditions, time-domain statistics, frequency-domain spectra, and envelope analysis have shown strong capability in identifying defect-related resonance responses [5,6]. These methods rely on the principle that localized defects generate impulsive forces that excite structural resonances, which can be revealed through demodulated vibration signals. However, the direct application of these approaches to PMDC motors presents practical difficulties specific to bearing diagnosis.

The brush-commutator interaction in PMDC motors produces strong broadband and non-stationary vibration whose magnitude varies directly with load and speed [7]. This motor-local excitation dominates the measured vibration signal, masking the comparatively weak resonance response generated by early bearing defects. Under such conditions, commonly used amplitude-sensitive indicators such as Root Mean Square (RMS) and kurtosis no longer reflect bearing condition but instead track operating variability [8]. This behavior is termed amplitude bias, which describes the condition where diagnostic features correlate more strongly with load-dependent vibration energy than with invariant bearing fault signatures.

Advanced data-driven approaches, particularly deep learning models applied directly to raw vibration signals, have achieved high accuracy on controlled benchmarks. However, their performance degrades under operating conditions absent from training data, their computational demands limit edge deployment, and their black-box nature obscures physical interpretability [9,10,11]. Critically, when amplitude bias is present, these models risk learning load-dependent amplitude patterns rather than invariant bearing fault characteristics.

A key physical observation in compact PMDC drivetrains is that vibration is not uniformly distributed across the structure. Measurements taken directly on the motor housing for bearing monitoring are strongly influenced by source-localized excitation, including commutation effects and rotor dynamics [12,13]. In contrast, a rigidly coupled downstream component primarily measures vibration that has propagated through the mechanical transmission path. The response at this location is governed by the transmissibility between the bearing fault location and the measurement point [14,15]. Consequently, the downstream location tends to attenuate non-propagating, motor-localized noise while remaining sensitive to structurally transmitted energy associated with bearing defects, particularly when the sensor is placed near the load path.

This spatial disparity provides an opportunity to distinguish local motor excitation from transmitted bearing fault responses; an opportunity not exploited in conventional single-sensor methods nor in multi-sensor fusion strategies that treat all channels symmetrically. This work investigates how this physical property of the drivetrain can be leveraged to construct a diagnostic representation that is inherently less sensitive to load-induced amplitude variations in bearing fault detection. Rather than increasing model complexity, the study develops a compact set of physically meaningful features derived from resonance-band energy and cross-sensor interaction metrics.

## 2. Literature Review

Robust bearing fault diagnosis under variable operating conditions remains a persistent challenge in rotating machinery research. Many established diagnostic methods are evaluated under relatively stable load and speed regimes, where defect-induced structural resonances dominate the measured vibration signal. Under such conditions, time-domain statistics [8,16], envelope analysis [17,18], and time–frequency transforms [19] have demonstrated reliable detection of localized bearing defects.

However, studies examining performance under varying load or speed report reduced robustness of amplitude-based indicators. Under changing operating conditions, envelope spectra and resonance-band energy often fluctuate strongly with load or speed, and defect-related modulation components can be partly masked by other excitation sources and sidebands [20]. Similarly, Yang et al. observed that common time-domain statistics, such as RMS and crest factor, correlate more strongly with operating conditions than with early-stage bearing degradation in electric motor drives [21]. These findings suggest that amplitude-based features can be highly sensitive to operating-condition variation in motor–bearing systems, limiting their reliability for bearing diagnosis for PMDC applications unless such variability is explicitly accounted for.

To mitigate the limitations of handcrafted descriptors, deep learning models have been applied directly to vibration signals. Tong et al. [22] reported 99.2% classification accuracy on the CWRU dataset using a deep neural network, while recent Transformer-based architectures [23], including lightweight TSL-Transformer and HEMA-Transformer variants, achieve comparable performance on CWRU, with reported accuracies around 98–99%. These results indicate that deep networks can learn highly discriminative fault representations when training and testing samples are drawn from similar operating regimes. However, their practical deployment faces a tripartite challenge: (1) computational expense that hinders edge deployment [24]; (2) interpretability barriers that obscure the physical fault mechanism [25]; and, most critically, (3) susceptibility to amplitude bias.

Cross-load experiments reported in [26] demonstrate significant accuracy degradation when models trained under one operating condition are evaluated under another. Such results suggest that learned representations may encode operating-condition–dependent signal characteristics. Domain adaptation methods have been proposed to address distribution mismatch [27]; however, these approaches typically introduce additional alignment mechanisms and require access to target-domain data during adaptation, increasing system complexity and limiting suitability for embedded PMDC monitoring.

Recognizing the limitations of single-sensor analysis, several studies have explored multi-sensor fusion for improved robustness [28,29]. Also, fusion architectures that concatenate or jointly encode vibration signals from multiple accelerometers generally report incremental improvements over single-sensor baselines [30]. More recently, cross-attention–based fusion networks have been applied to multi-channel vibration signals, demonstrating improved separation between normal and faulty bearings by modeling inter-channel correlations [31]. However, in these approaches, sensors are treated symmetrically; each channel contributes to the fused representation without explicit differentiation between motor-local excitation sources and downstream transmission-path measurements.

Physics-informed approaches [32,33] have also been proposed. Lu et al. introduced a physics-informed feature-weighting method in which prior knowledge of characteristic bearing fault frequencies is embedded into a CNN through a dedicated weighting layer and associated regularization term, thereby improving interpretability by emphasizing fault-related spectral components [34]. However, this strategy does not explicitly address load-induced amplitude sensitivity, because the underlying representation remains dominated by amplitude variations in the envelope or spectral domain [34].

What remains insufficiently addressed in the literature is a diagnostic framework that explicitly models the excitation–transmission distinction inherent in PMDC motor systems. While transmissibility-based techniques are well established in structural health monitoring for damage localization [35,36], their systematic integration into compact feature representations for bearing fault diagnosis in systems where the fault source and dominant excitation are collocated remains limited. In particular, prior studies have not explicitly used a downstream sensor solely as a conditioning reference for motor-side resonance features, nor have they selected resonance bands based on both fault separability and transmission stability.

To address this, this work proposes the CREA framework. CREA circumvents direct dependence on excitation amplitude by adopting a physics-guided, transmission-aware resonance analysis. Rather than explicitly tracking kinematic fault frequencies, the framework focuses on the fault-modulated energy contained within empirically selected structural resonance bands, which provides a more robust diagnostic indicator under variable operating conditions.

The proposed “attention” mechanism is a lightweight, deterministic signal-processing operation, not a learned neural attention model. It leverages a vibration signal from a reference sensor to compute interaction metrics that condition features extracted from a source sensor. Through this process, CREA explicitly decouples local, load-dependent excitation effects from fault-related energy that propagates through the mechanical transmission path.

To this end, the contributions of this work are as follows:

- A physics-guided, reference-conditioned envelope representation. A downstream vibration sensor is used exclusively to condition motor-side resonance features based on transmissibility and modulation consistency rather than as an additional diagnostic channel.
- It proposes, for the tested PMDC assembly, a transmission-informed carrier band selection procedure based on dual criteria of condition separability and cross-sensor transmission stability, without relying on bearing characteristic frequencies.
- It demonstrates, through controlled normalization ablation, that conventional amplitude-based vibration features achieve high apparent accuracy by encoding run-specific amplitude patterns rather than true fault excitation, exposing a critical hidden failure mode in vibration-based diagnosis for bearings.

## 3. Materials and Methods

### 3.1. Framework Overview

**Figure 1:**
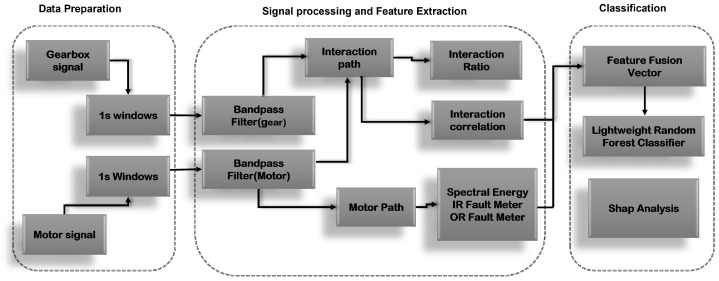
Schematic overview of the Cross-Reference Energy Attention (CREA) framework.

The proposed CREA framework implements a physics-informed diagnostic approach designed to maintain high classification fidelity within the computational constraints of industrial edge hardware. As illustrated in Figure 1, the architectural workflow initiates with the simultaneous acquisition of vibration signatures from motor-side and gearbox-side locations. Crucially, these dual measurement streams are processed independently during the initial stages to preserve their distinct spatial characteristics.

Time-synchronous segmentation and band-limited filtering are applied separately to each channel, ensuring temporal alignment and signal consistency required for subsequent inter-sensor analysis. Following this parallel pre-processing, the demodulated signal envelopes are analyzed within the CREA Feature Engine, where inter-sensor interaction metrics, specifically correlation and energy-ratio measures, are computed to attenuate localized, load-dependent noise.

The resulting feature vector is subsequently refined through a physics-informed feature selection protocol and passed to a lightweight Random Forest classifier for final fault diagnosis.

### 3.2. Experimental Testbed and Data Acquisition

**Figure 2:**
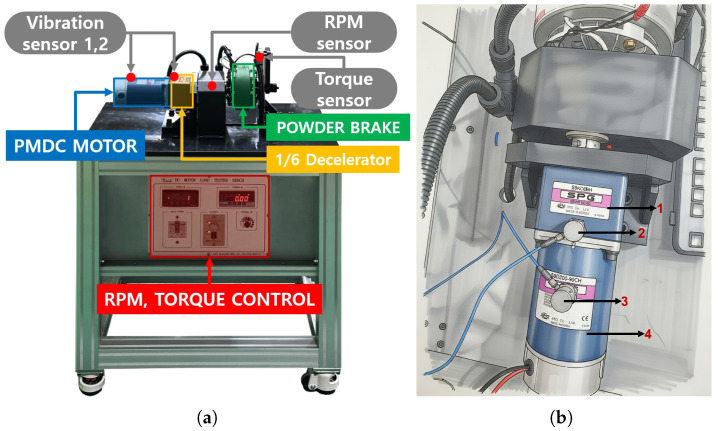
Experimental testbed: (**a**) ALPS dynamometer system setup; (**b**) sensor placement and measurement locations. In (**b**) 1. Gearbox casing; 2. Vibration sensor 2; 3. Vibration sensor 1; 4. PMDC motor.

The experiment was conducted at the Defence and Reliability Laboratory, Kumoh National Institute of Technology, Gumi, Republic of Korea, using an ALPS dynamometer system. The complete testbed layout, including the motor–gearbox assembly and dynamometer brake, is shown in Figure 2a. The actuation unit consists of a 90 V, 1/4 HP brushed DC motor with a no-load speed of 2760 RPM coupled to a 6:1 reduction gearbox. This configuration is representative of industrial conveyor and robotic drive systems operating under moderate speed and load conditions.

To emulate non-stationary industrial operation, the drivetrain was subjected to a controlled load-variation protocol using an ALPS magnetic particle brake (ALPS Technical Services, Abu Dhabi, United Arab Emirates). For each bearing condition, vibration data were acquired in discrete experimental runs, each corresponding to approximately 10 min of continuous operation. Within each run, the applied load was increased in five successive 2 min intervals by adjusting the POWDER_VR input, resulting in torque levels ranging from approximately 0.1 Nm to 1.3 Nm, as summarized in Table 1. This stepwise loading introduces natural speed fluctuations and variable mechanical resistance within each run, producing realistic non-stationary operating conditions while preserving a well-defined batch structure for subsequent analysis.

To capture vibration signatures at both the excitation source and along the transmission path, the system was instrumented with two NI 9234 piezoelectric accelerometers (Emerson, Seoul, Republic of Korea), as illustrated in Figure 2b. The motor-side sensor (labeled 3) was mounted on the motor housing to measure vibration associated with bearing excitation and local structural response at the source. A second sensor (labeled 2) was mounted on the gearbox casing to capture vibration energy transmitted through the drivetrain. Both channels were sampled synchronously at 2000 Hz using a National Instruments cDAQ-9174 chassis (Emerson, Seoul, Republic of Korea). This sampling rate was selected to ensure adequate spectral resolution and bandwidth coverage for mid-frequency resonance analysis, while maintaining synchronized acquisition across both sensor locations.

Three bearing health states were evaluated: healthy (baseline), inner-race (IR) fault, and outer-race (OR) fault. To emulate realistic early-stage localized defects in a repeatable manner, seeded faults were introduced on the drive-end bearing using controlled precision cutting. A narrow, localized linear groove was carefully introduced on either the inner race or the outer race using a handheld electric angle grinder equipped with a thin abrasive cut-off disc.

**Figure 3:**
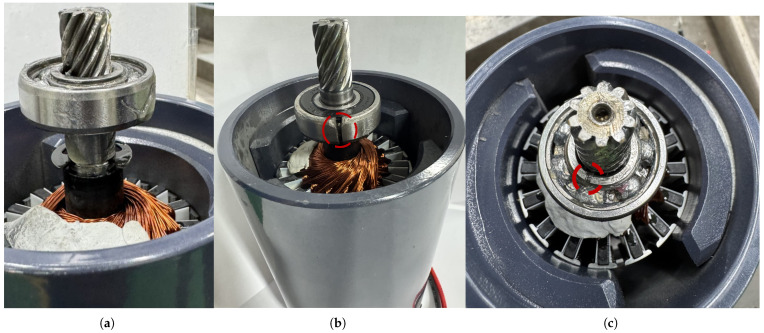
Bearing conditions investigated in this study: (**a**) healthy bearing with intact raceway surface; (**b**) inner-race (IR) bearing with a localized linear groove defect (highlighted); (**c**) outer-race (OR) bearing with a localized linear groove defect (highlighted).

Figure 3 illustrates the three bearing conditions investigated in this study. The healthy bearing exhibits an intact and continuous raceway surface, whereas the faulty bearings show clearly identifiable localized groove defects on the inner race and outer race, respectively. The marked regions in Figure 3b,c highlight the intentionally introduced defect locations, confirming that the damage is spatially localized rather than circumferential.

Such localized crack-like defects are representative of early-stage fatigue mechanisms, including subsurface crack initiation and localized surface spalling. During operation, rolling-element contact with these defects generates impulsive excitations that give rise to modulated vibration responses characteristic of localized bearing faults. This seeded-fault approach is widely adopted in bearing diagnostics research to enable controlled and repeatable experimentation without artificially exaggerating fault severity [12].

### 3.3. Signal Conditioning and Windowing Strategy

The raw vibration signals sampled at 2000 Hz were subjected to a consistent conditioning pipeline. Transient segments associated with load application and release were excluded in order to isolate steady-state behavior within each torque interval.

The remaining signals were segmented into fixed-length windows of 1-s duration with 50% overlap as summarized in Table 2. This window length was selected to balance temporal resolution and frequency resolution, providing sufficient spectral fidelity for mid-frequency resonance analysis while maintaining an adequate number of samples for statistical evaluation.

Each window was assigned metadata labels, including bearing condition and experimental run identifier. These run identifiers were explicitly preserved and enforced during validation to prevent data leakage. All windows originating from a given experimental run were confined exclusively to either the training or testing partition. This strategy ensures that the reported classification performance reflects generalization to unseen operating conditions rather than memorization of run-specific amplitude or operating artifacts.

### 3.4. Frequency-Domain Analysis and Carrier Band Definition

The objective of this subsection is to define a generalized, physics-informed framework for identifying optimal carrier bands for envelope analysis. While specific resonance frequencies vary across motor-gearbox assemblies due to differences in mass, stiffness, and operating speeds, the physical principles of fault transmission remain consistent. Therefore, rather than assuming a priori fault frequencies or fixing a static bandwidth, we propose a transferable selection procedure based on two criteria: (1) Transmission Stability (ensuring the signal propagates through the structure) and (2) Spectral Separability (ensuring the band contains fault-discriminative energy).

To implement this strategy, the raw vibration signals were first transformed into the frequency domain. Let x(n) denote a zero-mean vibration signal segment of length *N*. The single-sided magnitude spectrum was computed as:

(1) $$A\left(f_{k}\right)=\frac{2}{N}\left|\sum_{n=0}^{N-1}x\left(n\right)\;e^{-j2\pi kn/N}\right|,$$

where $f_{k}=kf_{s}/N$ , $f_{s}$ is the sampling frequency, and *k* denotes the frequency-bin index. To suppress stochastic variability, magnitude spectra were averaged across multiple segments for the motor-side signal $A_{m}\left(f\right)$ and the gearbox-side signal $A_{g}\left(f\right)$ .

The adaptive selection protocol proceeds in two stages:

Stage 1: Transmission Profiling

To identify frequency regions where mechanical energy propagates consistently through the drivetrain, a frequency-domain transmission metric was defined. Transmission profiling was performed using spectra averaged under the Normal condition only, such that the resulting metric reflects structural propagation characteristics independent of fault excitation.

The magnitude transmissibility was computed as

(2) $$H\left(f\right)=\frac{A_{g}\left(f\right)}{A_{m}\left(f\right)+\varepsilon },$$

where $\varepsilon =10^{-6}$ is a small regularization constant introduced to prevent numerical instability when $A_{m}\left(f\right)$ approaches zero.

For any candidate band $B=[f_{L},f_{H}]$ , a band-averaged transmission index $T_{B}$ was calculated as

(3) $$T_{B}=\frac{1}{\left|B\right|}\int_{f_{L}}^{f_{H}}H\left(f\right)\;df,$$

where $\left|B\right|=f_{H}-f_{L}$ .

This metric identifies structural passbands where vibration energy is consistently transmitted from the bearing location to the gearbox sensor, while suppressing frequency regions dominated by localized motor excitation.

Stage 2: Separability Scanning

Within bands exhibiting acceptable transmission consistency, discriminative relevance was quantified using motor-side spectral energy.

For each operating condition c∈{Normal,IR,OR} ,

(4) $$E_{B}\left(c\right)=\frac{1}{\left|B\right|}\int_{f_{L}}^{f_{H}}A_{m}^{2}\left(f\right)\;df.$$

The relative inter-class separation metric was defined as

(5) $$S_{B}=\frac{\left|E_{B}\left(\mathrm{IR}\right)-E_{B}\left(\mathrm{Normal}\right)\right|+\left|E_{B}\left(\mathrm{OR}\right)-E_{B}\left(\mathrm{Normal}\right)\right|}{2E_{B}\left(\mathrm{Normal}\right)}.$$

Carrier-band selection was therefore formulated as a constrained optimization problem: maximize $S_{B}$ subject to acceptable transmission consistency $T_{B}$ .

#### Application to Experimental Platform

For this study, the adaptive protocol was implemented by defining a search space of mid-frequency regions. Based on the initial transmission profiling, the following candidate bands were defined for evaluation:

$$B_{i}\in \left\{\left[150,400\right],\left[200,500\right],\left[300,600\right],\left[400,800\right],\left[200,800\right]\right\}\;\mathrm{Hz}.$$

**Figure 4:**
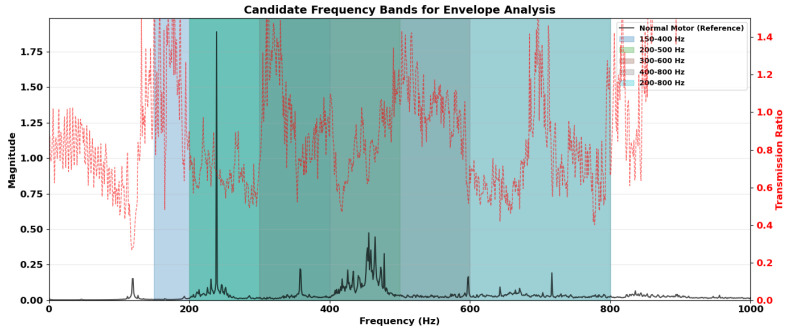
Averaged motor-side vibration spectrum with evaluated mid-frequency candidate carrier bands highlighted.

These bands were specifically selected to exclude low-frequency regions dominated by speed-dependent harmonics and electromagnetic effects, while encompassing frequency ranges commonly associated with structural resonance responses excited by localized bearing defects. This search strategy is consistent with classical envelope analysis theory, in which defect-induced impacts excite structural resonances rather than appearing directly at characteristic fault frequencies. The evaluated candidate bands are illustrated in Figure 4, overlaid on the averaged motor-side vibration spectrum.

The final carrier band ( 300–600Hz ) reported in the Section 4 corresponds to the band that maximized the separation metric $S_{B}$ while satisfying transmission consistency criteria.

This selection procedure is transferable: the same transmission-separation evaluation can be recomputed for alternative motor powers, gearbox transmission ratios, or bearing models to identify their respective optimal carrier bands.

### 3.5. Physics-Guided Feature Construction

The final carrier band, as observed between the 300–600 Hz in Figure 4, was further refined into fault-specific audit sub-bands. Inspection of the motor-side spectrum under each fault condition revealed localized resonant peaks: near 360 Hz for inner-race (IR) faults and near 515 Hz for outer-race (OR) faults. To confirm the diagnostic relevance of these peaks, the separation metric $S_{B}$ was computed for narrow bands around each candidate peak. The 340–380 Hz band centered on the IR peak and the 480–550 Hz band centered on the OR peak yielded the highest separation from the normal condition. Consequently, these sub-bands were designated as $E_{\mathrm{IR}}$ and $E_{\mathrm{OR}}$ to capture fault-modulated resonance energy with maximal class separability. It is important to note that the second sensor signal, i.e., the gearbox-side signal, is not used as a direct diagnostic source. Instead, it is used exclusively to compute interaction metrics that condition motor-side features based on physically consistent vibration transmission through the drivetrain.

Let $a_{m}\left(t\right)$ and $a_{g}\left(t\right)$ denote the windowed motor-side and gearbox-side vibration signals, respectively. Both signals were filtered using a fourth-order zero-phase Butterworth band-pass filter with passband;

(6) B=[300,600]Hz,

such that

(7) $$a_{m}^{B}\left(t\right)={\mathrm{BP}}_{300–600}\left\{a_{m}\left(t\right)\right\},\;a_{g}^{B}\left(t\right)={\mathrm{BP}}_{300–600}\left\{a_{g}\left(t\right)\right\},$$

where forward–backward filtering was applied to eliminate phase distortion. This conditioning isolates the resonance-dominated frequency region identified in Section 3.3 while keeping low-frequency commutation components and high-frequency noise in check.

Fault-related excitation was quantified using motor-side spectral energy computed within two empirically identified audit sub-bands located inside the carrier band. Let $A_{m}^{B}\left(f\right)$ denote the magnitude spectrum of $a_{m}^{B}\left(t\right)$ . The carrier-band energy features were defined as

(8) $$E_{\mathrm{IR}}=\int_{340}^{380}\left|A_{m}^{B}\left(f\right)\right|\;df,\;E_{\mathrm{OR}}=\int_{480}^{550}\left|A_{m}^{B}\left(f\right)\right|\;df.$$

These features capture resonance-amplified vibration energy induced by localized defect impacts, without reliance on explicit bearing characteristic frequencies. Energy integration over bounded frequency intervals is widely used to obtain robust indicators under variable speed and load conditions.

Furthermore, two interaction features were computed using both filtered signals. A band-limited transmission ratio was defined as

(9) $$R_{T}=\frac{\mathrm{RMS}\;\left(a_{g}^{B}\left(t\right)\right)}{\mathrm{RMS}\;\left(a_{m}^{B}\left(t\right)\right)},$$

which measures the fraction of motor-side vibratory energy transmitted to the gearbox casing through the mechanical path.

In addition, modulation synchrony between sensors was quantified using envelope correlation. Hilbert envelopes were first computed as

(10) $$e_{m}\left(t\right)=\left|a_{m}^{B}\left(t\right)+j\;H\left\{a_{m}^{B}\left(t\right)\right\}\right|,\;e_{g}\left(t\right)=\left|a_{g}^{B}\left(t\right)+j\;H\left\{a_{g}^{B}\left(t\right)\right\}\right|,$$

and the envelope correlation was then defined as

(11) $$\rho =\frac{E\;\left[\left(e_{m}-\mu_{m}\right)\left(e_{g}-\mu_{g}\right)\right]}{\sigma_{m}\sigma_{g}},$$

where μ and σ denote the mean and standard deviation, respectively. This metric reflects the temporal coherence of amplitude modulation between the motor and gearbox rather than excitation strength.

For comparison purposes, conventional statistical features such as RMS, kurtosis, and skewness were extracted from the unfiltered motor-side signal $a_{m}\left(t\right)$ . These features were included solely as an amplitude-sensitive baseline for ablation analysis and are not intended to represent physics-guided descriptors. The final physics-guided feature vector, therefore, consists of four features: two carrier-band energy features ( $E_{\mathrm{IR}}$ , $E_{\mathrm{OR}}$ ) and two cross-sensor interaction features ( $R_{T}$ , ρ ).

### 3.6. Validation and Evaluation Protocol

To evaluate generalization across independent acquisition runs, grouped cross-validation was implemented using GroupKFold with five splits. Unlike standard *k*-fold cross-validation, which randomly partitions samples, this protocol groups data by experimental run identifier $g_{i}$ . All windows originating from a given run were assigned exclusively to either the training or testing partition within each fold. This grouping prevents the classifier from exploiting run-specific amplitude scaling or background characteristics unrelated to the underlying fault mechanism.

The diagnostic task was formulated as a three-class classification problem corresponding to the health states: Normal, Inner-Race (IR), and Outer-Race (OR). Performance was evaluated at the window level, where each 1s segment was classified independently.

To assess the influence of amplitude bias, a controlled ablation study was conducted. For the baseline configuration, conventional amplitude-sensitive features were normalized on a per-run basis according to

(12) $$x_{\mathrm{norm}}^{\left(r\right)}=\frac{x^{\left(r\right)}-\mu^{\left(r\right)}}{\sigma^{\left(r\right)}},$$

where $\mu^{\left(r\right)}$ and $\sigma^{\left(r\right)}$ denote the mean and standard deviation computed within run *r*. This operation removes run-dependent amplitude scaling while preserving intra-run structure. In contrast, the physics-guided CREA features were left unnormalized because they are derived from band-limited spectral structure and cross-sensor interaction metrics rather than from absolute signal magnitude.

Classification was performed using a Random Forest model with fixed hyperparameters: n_estimators = 100, max_depth = 10, criterion = ’gini’, min_samples_split = 2, min_samples_leaf = 1, and random_state = 42. Hyperparameters were selected prior to evaluation and held constant across all feature configurations. No tuning was performed on test partitions. By maintaining an identical classifier configuration across all experiments, observed performance differences reflect differences in feature representation rather than model capacity or optimization.

#### Sensor Placement Sensitivity

To assess the influence of gearbox-side sensor placement on the cross-sensor interaction features, a controlled relocation experiment was conducted in addition to the primary dataset.

Two gearbox accelerometer configurations were evaluated:

- G0: Original gearbox-mounted location used throughout the primary dataset.
- G2: Relocated configuration in which the gearbox sensor was repositioned relative to the motor–gear assembly.

All other experimental parameters were held constant, including motor configuration, bearing condition, torque profile, sampling frequency (2000 Hz), segmentation scheme (1 s windows with 50% overlap), and the feature extraction procedure defined in Section 3.4.

Data were acquired under two bearing states: Normal and outer-race (OR) fault. Feature computation employed identical 300–600 Hz band-pass filtering and the interaction metrics transmission ratio $R_{T}$ and envelope correlation ρ .

To quantify relative class contrast and placement dependence, two descriptive indices were computed:

Separability Index

(13) $$S=\frac{\left|\mu_{\mathrm{Normal}}-\mu_{\mathrm{OR}}\right|}{\mu_{\mathrm{Normal}}}\times 100\%$$

Placement Sensitivity Index

(14) $$\Delta =\frac{\left|\mu_{G0}-\mu_{G2}\right|}{\mu_{G0}}\times 100\%$$

where μ denotes the window-level mean of the corresponding feature.

Because the sensitivity dataset consisted of a single acquisition per condition per placement, the analysis was restricted to feature-level distribution comparison.

## 4. Results

Experimental results from the CREA framework are presented as follows: (1) frequency- domain characterization and empirical carrier-band selection; (2) statistical distributions of window-level features; (3) diagnostic performance under-run-stratified GroupKFold validation, including an ablation test for amplitude dependence; (4) interpretability analysis via SHAP to quantify feature contributions.

### 4.1. Frequency-Domain Characterization and Carrier Band Selection

**Figure 5:**
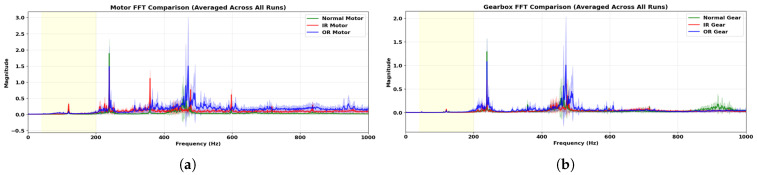
Averaged magnitude spectra for (**a**) motor-side and; (**b**) gearbox-side vibration signals under Normal (N), Inner-Race (IR), and Outer-Race (OR) conditions. The shaded yellow region highlights the low-frequency band where substantial spectral overlap occurs across conditions.

Figure 5 shows the averaged magnitude spectra of the motor-side and gearbox-side vibration signals across all runs and operating conditions. The dominant spectral components occur at similar frequency locations for both sensing points, with differences primarily in magnitude. Below approximately 200 Hz, the spectra of the three bearing conditions overlap substantially, providing limited condition separability.

This low-frequency region is dominated by speed-dependent components and brush–commutator excitation, which introduce broadband and non-stationary vibration unrelated to bearing condition. The dense spectral content in this range reduces the effective diagnostic signal-to-noise ratio at the motor housing. In contrast, clearer differences between conditions appear within the mid-frequency region, where defect-induced impacts excite structural resonances that are less affected by commutation-related disturbances.

**Figure 6:**
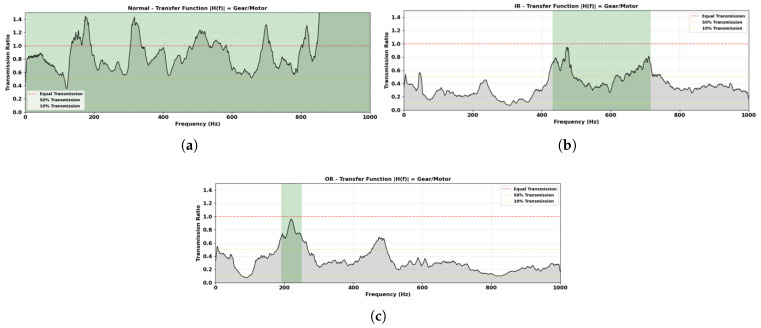
Magnitude transfer function |H(f)| between gearbox-side and motor-side spectra under (**a**) Normal (N); (**b**) Inner-Race (IR), and (**c**) Outer-Race (OR) conditions.

Figure 6 presents the magnitude transfer function between the gearbox-side and motor-side spectra computed using Equation (2). The transmission ratio varies with frequency and differs across operating conditions. Under the Normal condition Figure 6a, a relatively broad transmission is observed across the mid-frequency range. The IR condition Figure 6b exhibits higher transmission primarily within approximately 400–700 Hz. The OR condition Figure 6c shows elevated transmission concentrated around 180–250 Hz. The shaded green regions highlight frequency intervals exhibiting comparatively stronger transmission between the sensing locations.

As listed in Table 3, the candidate bands exhibit different transmission and separation characteristics.

Among the evaluated bands, the 300–600 Hz range achieved the highest separation metric Equation (5) while maintaining stable transmission between the motor and gearbox sensors. Importantly, this selection was performed without referencing bearing characteristic defect frequencies, ensuring that the carrier band was determined solely from empirical transmission and separation behavior.

### 4.2. Window-Level Feature Distributions

**Figure 7:**
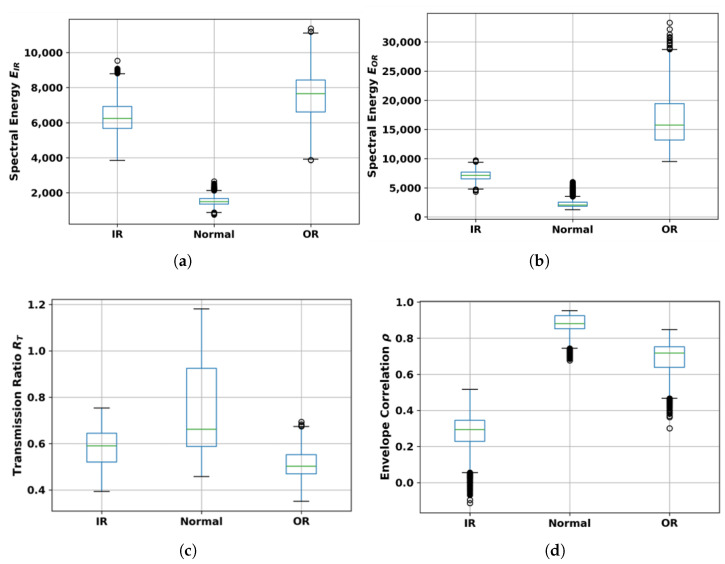
Distribution of window-level CREA features across bearing conditions. Boxes represent the interquartile range (25th to 75th percentile) with the median as a horizontal line; whiskers extend to 1.5× IQR; points beyond are outliers. (**a**) $E_{\mathrm{IR}}$ : integrated spectral magnitude in 340–380 Hz (proportional to vibration energy). (**b**) $E_{\mathrm{OR}}$ : integrated spectral magnitude in 480–550 Hz (proportional to vibration energy). (**c**) $R_{T}$ : RMS transmission ratio (dimensionless). (**d**) ρ : envelope correlation coefficient (dimensionless, range −1 to 1).

Figure 7 presents box plots of the four CREA features across the three bearing conditions. In each plot, the central line indicates the median, box edges mark the 25th and 75th percentiles (IQR), whiskers extend to 1.5× IQR, and points beyond are outliers.

The spectral energy values ( $E_{\mathrm{IR}}$ , $E_{\mathrm{OR}}$ ) are computed as Equation (8) and have units of g·Hz.

For $E_{\mathrm{IR}}$ , the median value for the inner race condition is higher than for the Normal and outer race conditions. For $E_{\mathrm{OR}}$ , the median value for the outer race condition is higher than for the inner race and Normal conditions.

For $R_{T}$ (dimensionless) Equation (9), the median value is highest for the outer race condition, intermediate for Normal, and lowest for the inner race condition. For ρ (dimensionless, range −1 to 1) Equation (11), the median value is highest for the inner race condition and lower for Normal and outer race conditions.

### 4.3. Ablation of Diagnostic Representation Components Under GroupKFold Validation

**Figure 8:**
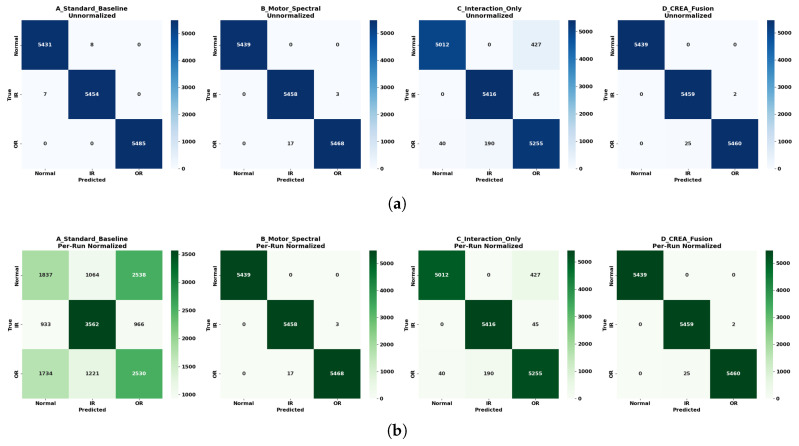
Confusion matrices comparing feature strategies. (**a**) Classification results using unnormalized window features. (**b**) Classification results after applying per-run normalization to the baseline features.

Figure 8 presents the window-level confusion matrices obtained from five-fold run-stratified GroupKFold validation, where windows from the same acquisition run were assigned to the same fold. In each confusion matrix, diagonal entries represent correctly classified windows, while off-diagonal entries indicate misclassifications. For example, a window from the Inner-Race condition classified as Outer-Race would appear as an off-diagonal entry in the IR row under the OR column.

Two evaluation settings are shown: (a) using original window features and (b) using fold-local normalization applied to assess sensitivity to amplitude scaling. Without fold-local normalization, all feature configurations exhibit dominant diagonal entries, indicating strong class separability. After normalization, the Standard Motor Baseline shows substantial degradation, with increased off-diagonal assignments across all classes. In contrast, the carrier energies and CREA feature sets maintain near-diagonal confusion patterns under the same protocol. Table 4 summarizes window-level accuracy, Macro-F1, and balanced accuracy under run-stratified GroupKFold evaluation with fold-local normalization.

An extended stepwise ablation was performed using the same configuration to evaluate all single, dual, triple, and full combinations of the four CREA features. Table 5 summarizes the window-level accuracy (mean ± standard deviation), Macro-F1, and balanced accuracy for each configuration.

Among single-feature models, spectral OR achieved the highest window accuracy, while the transmission ratio alone produced the lowest performance. Dual carrier energy combinations achieved near-perfect accuracy. The inclusion of envelope correlation increased Macro-F1 relative to carrier energy alone. Across all classes, the carrier-band spectral energy features span the widest SHAP ranges, whereas the interaction features span97 window accuracy with balanced class performance.

### 4.4. Feature Contribution Analysis via SHAP

SHAP analysis was performed on the Random Forest model trained using the CREA fusion feature set to quantify the contribution of each feature to the classifier output.

**Figure 9:**
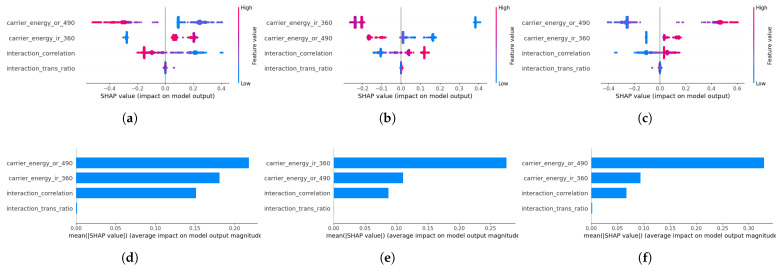
SHAP-based interpretation of CREA features across classes. (**a**–**c**) SHAP summary plots for the three classes (Healthy, IR, OR). (**d**–**f**) Mean absolute SHAP values for the same classes, quantifying overall feature importance.

Figure 9a–c shows the class-wise SHAP beeswarm plots for healthy, IR, and OR conditions. Across all classes, the carrier-band spectral energy features span the widest SHAP ranges, whereas the interaction features span narrower ranges centered closer to zero. Figure 9d–f presents the mean absolute SHAP values aggregated across all samples. The feature ranking is consistent across classes.

### 4.5. Robustness of Interaction Features to Sensor Placement

**Figure 10:**
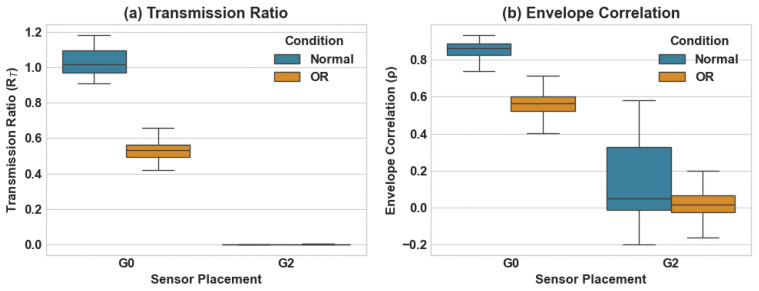
Interaction feature distributions under different gearbox sensor placements. (**a**) Transmission ratio $R_{T}$ . (**b**) Envelope correlation ρ .

Figure 10 presents the window-level distributions of the transmission ratio $R_{T}$ and envelope correlation ρ under placements G0 and G2 for the Normal and OR conditions.

Under G0, Healthy windows exhibit higher central tendency values of both interaction features relative to OR windows. Under G2, the absolute magnitude of both $R_{T}$ and ρ decreases substantially for both conditions.

Although absolute feature magnitudes decrease under G2, relative separation between Normal and OR conditions remains observable.

## 5. Discussion

The results establish a structured progression from spectral characterization to model-level attribution for bearing fault classification in the tested PMDC motor gear assembly. The frequency-domain analysis in Section 4.1 shows that spectral components below 200 Hz overlap substantially across Normal, Inner-Race, and Outer-Race conditions. This region is dominated by commutation harmonics and speed-dependent dynamics measured at the motor housing and does not provide reliable separability for bearing faults in this configuration. In contrast, clearer condition-dependent differences appear in the mid-frequency region. The 300–600 Hz band was selected because it maximized inter-class separation while maintaining stable cross-sensor transmission (Table 3). This selection reflects the resonance and structural transmission properties of this specific drivetrain and is not assumed to be transferable to other motor or bearing configurations. However, the key contribution lies not in the specific frequency band, but in the transferable selection procedure itself. The two-stage process, transmission profiling followed by separability scanning, is hardware-agnostic and will be recomputed. For a different motor size, gearbox ratio, or bearing type, the same procedure would identify the optimal carrier band for that specific configuration. The 300–600 Hz band reported here is therefore an empirical outcome of applying this method to our test rig, not a fixed recommendation.

Having fixed the carrier band, the window-level feature distributions in Figure 7 provide the first quantitative evidence of class structure. The spectral energy features show class-specific median shifts: $E_{\mathrm{IR}}$ increases predominantly for Inner-Race faults, whereas $E_{\mathrm{OR}}$ increases for Outer-Race faults. This indicates that the two defect types excite different resonant components within the selected band. In contrast, the interaction features $R_{T}$ and ρ show lower central values under both fault conditions than under Normal. This pattern indicates that bearing defects degrade transmission coherence between the motor and gearbox sensors, while healthy bearings preserve it. Thus, the box plots reveal two mechanisms: excitation localization, spectral energy, and transmission integrity interaction metrics.

The run-stratified GroupKFold validation further clarifies which of these mechanisms are robust to amplitude scaling. When evaluated without fold-specific normalization, the Standard Motor Baseline appears highly accurate. However, under run-stratified GroupKFold with fold-local normalization, its performance decreases to 0.495±0.110 (Table 4). Because windows from the same acquisition run are confined to a single fold, and normalization is performed independently within each fold, this degradation indicates that the baseline features depended on run-specific amplitude differences rather than on an invariant bearing-fault structure. In contrast, the spectral carrier features and the full CREA feature set maintain performance ( 0.999±0.002 ), consistent with the distribution-level separability observed in Figure 7. This shows that the discriminative structure of the carrier-band energies is not solely driven by global amplitude scaling.

The comprehensive ablation Table 5 provides a more detailed view of the internal hierarchy of the feature set. Single-feature results show strong asymmetry: the OR carrier energy alone achieves 0.987±0.016 , whereas the transmission ratio alone performs near chance level ( 0.503±0.272 ), and the envelope correlation alone reaches 0.951±0.061 . This indicates that, for the seeded faults and structural configuration tested, resonance-band excitation contains the dominant discriminatory information. When $E_{\mathrm{IR}}$ and $E_{\mathrm{OR}}$ are combined, accuracy increases to 0.999±0.002 , which is statistically indistinguishable from the full four-feature CREA model ( 0.99±0.002 ). Therefore, in this controlled dataset, the interaction features do not increase raw classification accuracy beyond what is already achieved by the spectral energies. Instead, they encode transmission characteristics that are partially redundant for separability under seeded conditions but structurally consistent with the physical interpretation suggested by the box plots.

The SHAP analysis confirms that the classifier exploits the same structure revealed by the distributions and ablation. For the inner race class, $E_{\mathrm{IR}}$ contributes positively to class prediction; for the outer race class, $E_{\mathrm{OR}}$ dominates. The interaction features exhibit smaller but consistent directional contributions, shifting predictions toward the Normal class when transmission coherence is preserved. This attribution pattern matches both the median shifts observed in Figure 7 and the hierarchy revealed in the extended ablation. Thus, the classification behavior can be traced directly to the excitation and transmission characteristics identified at the feature-distribution level, rather than to opaque model interactions.

The sensor placement experiment quantifies the sensitivity of the interaction metrics to structural coupling between the bearing location and the secondary sensor. As shown in Table 6, relocating the gearbox sensor from the rigid housing (G0) to the access cover (G2) reduces the mean transmission ratio for the Normal condition from 1.0300±0.0659 to 0.0088±0.0557 , and for the OR condition from 0.5293±0.0498 to 0.0026±0.0059 . A similar reduction is observed for the envelope correlation, where the Normal mean decreases from 0.8580±0.0445 at G0 to 0.1389±0.1992 at G2. These reductions indicate substantial attenuation of transmitted vibration energy at the G2 location.

Despite this attenuation, Table 7 shows that class separability remains measurable under G2, with separability values increasing to 70.5% for $R_{T}$ and 78.8% for ρ . This indicates that although absolute magnitudes decrease, the relative contrast between Normal and OR conditions persists. The increased standard deviation under G2, particularly for ρ , reflects reduced transmission consistency at the more compliant mounting location. These results demonstrate that the interaction metrics respond to changes in structural transmission path rather than exhibiting instability; however, reliable measurement requires placement on a mechanically continuous load path.

Taken together, the results show that, for the tested PMDC drivetrain with seeded bearing defects, classification performance is primarily resonance-driven, with spectral carrier energies providing the dominant separability. Interaction features do not materially improve accuracy in this dataset but remain physically interpretable descriptors of excitation propagation. The alignment between carrier-band selection, window-level separability, cross-validation behavior, comprehensive ablation, and SHAP attribution provides a consistent chain linking measured vibration structure to classification outcome without requiring high-capacity models.

## 6. Conclusions

This study investigated bearing fault classification in a PMDC motor–gear assembly using a dual-sensor vibration representation designed to distinguish resonance excitation from structural transmission behavior. Frequency-domain analysis identified a mid-frequency resonance region (300–600 Hz) that maximized inter-class separability while maintaining consistent cross-sensor transmission for the tested drivetrain. This carrier band was determined empirically from measured spectra and is specific to the structural characteristics of the experimental platform.

Window-level feature distributions demonstrated that spectral carrier energies encode fault-type–specific excitation, while transmission ratio and envelope correlation encode propagation coherence. Run-stratified GroupKFold evaluation showed that conventional amplitude-based baseline features collapse under fold-local normalization, indicating dependence on run-specific amplitude scaling. In contrast, the proposed carrier-band energies and interaction features maintain discriminatory structure under the same protocol.

The extended ablation, maintaining the same configurations, revealed that classification performance on this seeded-fault dataset is primarily resonance-driven, with spectral energies alone achieving near-perfect accuracy. Interaction features do not materially increase classification accuracy in this configuration but provide physically interpretable descriptors of excitation propagation. SHAP attribution confirms that the classifier relies predominantly on the resonance-band energies identified in the distribution analysis, with interaction features contributing directional influence consistent with transmission integrity.

Collectively, the results demonstrate that, for the tested PMDC drivetrain, bearing fault classification can be achieved using a small set of physically defined features without reliance on high-capacity models. The alignment among spectral selection, distribution-level separability, cross-validation performance, comprehensive ablation, and model attribution establishes a consistent link between the measured vibration structure and the predictive outcome.

### Limitations and Future Work

Several limitations constrain the interpretation of the present results and define directions for subsequent investigation.

The carrier band (300–600 Hz) was identified for a specific PMDC motor–gear assembly with seeded inner- and outer-race defects. The optimal resonance region is governed by the structural dynamics and transmission characteristics of this assembly and may therefore differ for motors with different power ratings, gearbox ratios, mounting conditions, or bearing geometries. Validation across multiple platforms is required to determine the extent to which the separability–transmission selection criterion remains effective under altered structural conditions.

A second limitation concerns the use of seeded defects under controlled laboratory conditions. Naturally evolving bearing degradation may produce weaker excitation, gradual progression, and different modulation behavior. Longitudinal studies on progressive fault development are necessary to evaluate early-stage sensitivity and to confirm that the observed feature separability persists under realistic wear mechanisms.

The dataset contained only bearing-related conditions and did not include non-bearing disturbances such as electrical anomalies, adjacent component vibration, or concurrent mechanical faults. Consequently, the ability of the transmission-based interaction metrics to discriminate bearing-induced excitation from unrelated vibration sources was not directly evaluated. Although the interaction features did not increase static classification accuracy in the present dataset, their intended role as transmission validators under mixed-fault conditions remains to be experimentally verified through controlled introduction of non-bearing disturbances.

The interaction metrics were also shown to be sensitive to secondary sensor placement. Relocation from a rigid gearbox housing to a more compliant access cover substantially attenuated both $R_{T}$ and ρ and increased variability, indicating reduced structural coupling. While class separability persisted under the tested relocation, insufficient coupling may degrade measurement stability. Systematic investigation of mounting stiffness, distance, and structural transfer characteristics across different assemblies is required to define practical deployment constraints.

Finally, only vibration signals from two accelerometers were considered. Integration of additional modalities, such as motor current or temperature, may enhance robustness under variable operating conditions. Multimodal fusion should therefore be evaluated to determine whether complementary sensing improves discrimination in more complex operational environments.

Future work will therefore focus on multi-platform validation, progressive fault monitoring, controlled mixed-disturbance testing to quantify the structural role of transmission metrics, systematic sensor placement studies, and multimodal fusion to enhance robustness under realistic operating conditions.

## Figures and Tables

**Table 1:** Load profile and torque levels under variable powder brake (VR) settings.

Time Segment	Powder VR Setting	Torque Range	Operating Condition
0–2 min	0%	0.1–0.3 Nm	Light load
2–4 min	20%	0.4–0.6 Nm	Low load
4–6 min	40%	0.6–0.9 Nm	Moderate load
6–8 min	60%	0.9–1.1 Nm	High load
8–10 min	80%	1.1–1.3 Nm	Peak load

**Table 2:** Summary of experimental dataset and windowing configuration.

Number of bearing conditions	3 (Healthy, IR, OR)
Runs per condition	5
Total experimental runs	15
Run duration	10 min
Window length	1 s
Window overlap	50%
Sampling frequency	2000 Hz
Total number of windows	16,385
Grouping unit for validation	run_id

**Table 3:** Frequency band evaluation based on transmission and separation metrics.

Band (Hz)	Center (Hz)	BW (Hz)	Transmission	Separation
150–400	275	250	0.528	0.8005
200–500	350	300	0.551	1.5596
300–600	450	300	0.565	4.9704
400–800	600	400	0.554	4.7871
200–800	500	600	0.537	2.3754

**Table 4:** Window-level classification performance under five-fold run-stratified GroupKFold validation with per-run normalization applied only to baseline features.

Feature Strategy	Features	Window Accuracy (Mean ± Std)	Macro-F1	Balanced Accuracy
Standard Motor Baseline	3	0.495±0.110	0.489	0.495
Carrier Energies (IR, OR)	2	0.999±0.002	0.999	0.999
Interaction ( $R_{T},\rho$ )	2	0.956±0.028	0.956	0.957
CREA Fusion (Energy + Interaction)	4	0.999±0.002	0.999	0.999

**Table 5:** Window-level classification performance across individual features and feature combinations.

Feature Strategy	Window Accuracy (Mean ± Std)	Macro-F1	Balanced Accuracy
Single Features			
Carrier Energy (IR)	0.777±0.223	0.594	0.777
Carrier Energy (OR)	0.987±0.016	0.619	0.987
Transmission Ratio $R_{T}$	0.503±0.272	0.264	0.503
Envelope Correlation ρ	0.951±0.061	0.487	0.951
Dual Feature Combinations			
IR + OR	0.999±0.002	0.999	0.999
IR + $R_{T}$	0.781±0.204	0.597	0.781
IR + ρ	0.988±0.038	0.797	0.988
OR + $R_{T}$	0.997±0.005	0.755	0.997
OR + ρ	0.994±0.022	0.932	0.994
$R_{T}$ + ρ	0.956±0.028	0.956	0.957
Triple Feature Combinations			
IR + OR + $R_{T}$	0.999±0.004	0.900	0.999
IR + OR + ρ	0.994±0.023	0.932	0.994
IR + $R_{T}$ + ρ	0.989±0.033	0.830	0.989
OR + $R_{T}$ + ρ	0.994±0.022	0.932	0.994
Full CREA—All Four Features	0.999±0.002	0.899	0.999

**Table 6:** Interaction Feature Statistics Under Sensor Placements.

Placement	Condition	$R_{T}$ (Mean ± Std)	ρ (Mean ± Std)	*n*
G0	Normal	1.0300 ± 0.0659	0.8580 ± 0.0445	1213
G0	OR	0.5293 ± 0.0498	0.5587 ± 0.0693	1249
G2	Normal	0.0088 ± 0.0557	0.1389 ± 0.1992	1127
G2	OR	0.0026 ± 0.0059	0.0294 ± 0.0871	1203

**Table 7:** Separability and placement sensitivity metrics (%).

Metric	$R_{T}$	ρ
Separability (G0)	48.6	34.9
Separability (G2)	70.5	78.8
Placement Sensitivity (Normal)	99.1	83.8
Placement Sensitivity (OR)	99.5	94.7

## Data Availability

The data presented in this study are available upon request from the corresponding author. The data are not publicly available due to laboratory regulations.
